# Supplementary material for: The 5α condensate state in 20Ne
Source: Nat Commun. 2023 Dec 11;14:8206. doi: 10.1038/s41467-023-43816-9 (PMC10713642; doi:10.1038/s41467-023-43816-9)
Supplement: Supplementary file 1 — Supplementary Information [file 41467_2023_43816_MOESM1_ESM.pdf]

Supplementary Figures

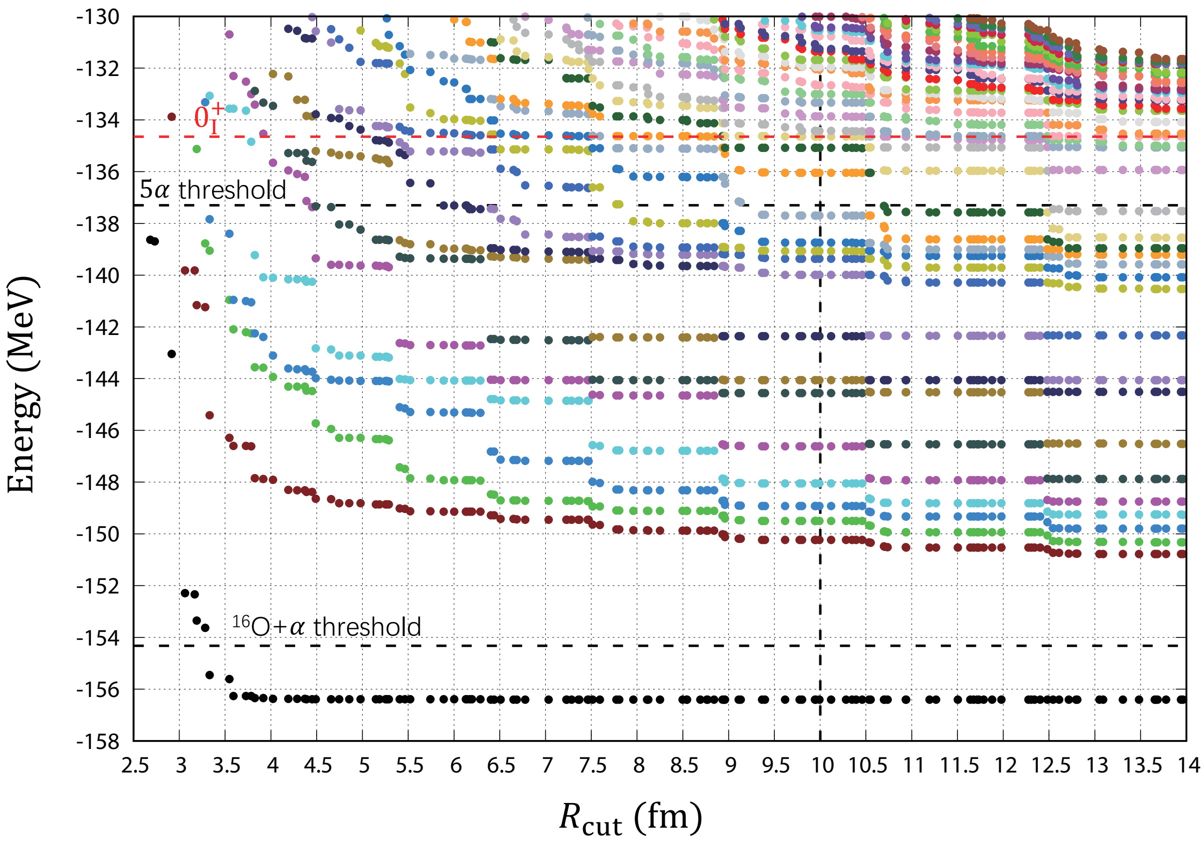

Supplementary Figure 1. **The dependence of energy eigenvalues in the GCM calculations on the radius cutoff parameter $R_{\rm cut}$.** The same color points represent the same λ_{th} eigenvalues. The two horizontal black dashed lines represent the $^{16}O + \alpha$ and 5α threshold, respectively. The obtained $0_{\rm I}^{+}$ state is also shown with the horizontal red dashed line. In the GCM calculations, the $R_{\rm cut}$ is chosen as 10 fm and it is marked in the vertical black dashed line.

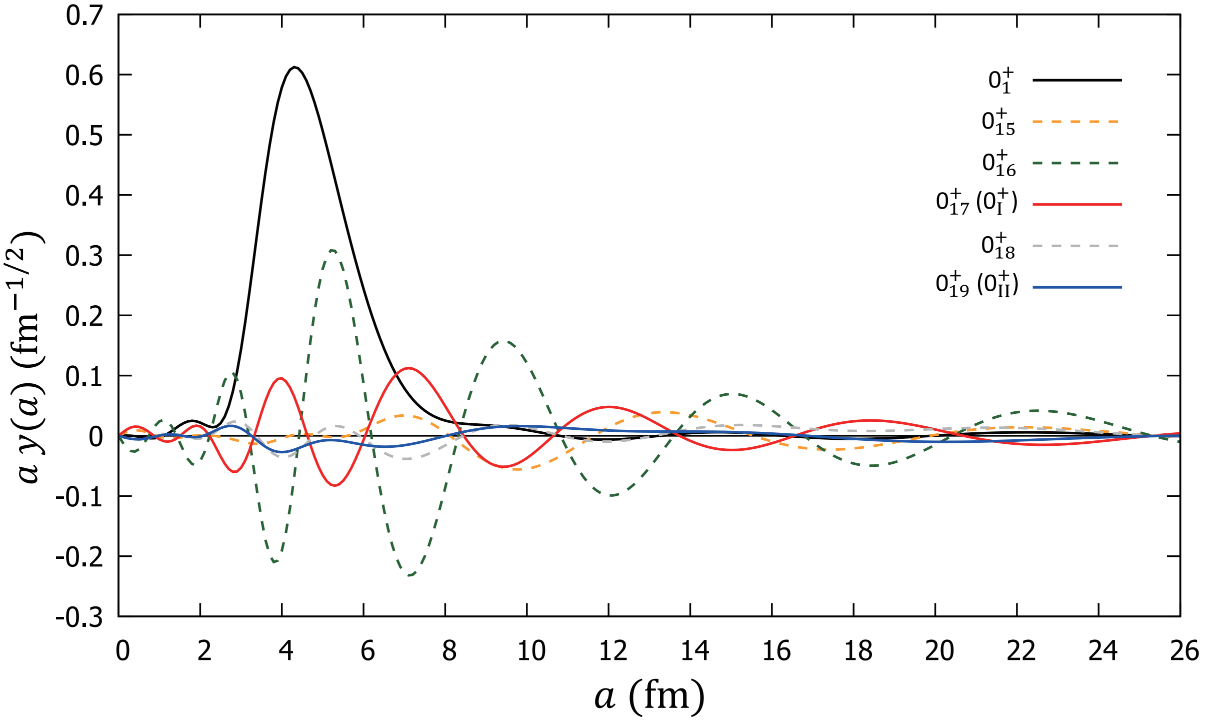

Supplementary Figure 2. The reduced width amplitudes of the ground state and excited states above 5α threshold in ^{20}Ne in the channel of ^{16}O (0_1^+) + α. The ground state 0_1^+, 0_{17}^+ ($0_{\rm I}^+$), and 0_{19}^+ ($0_{\rm II}^+$) states are shown in solid lines. The 0_{15}^+, 0_{16}^+, and 0_{18}^+ states are shown in dashed lines.

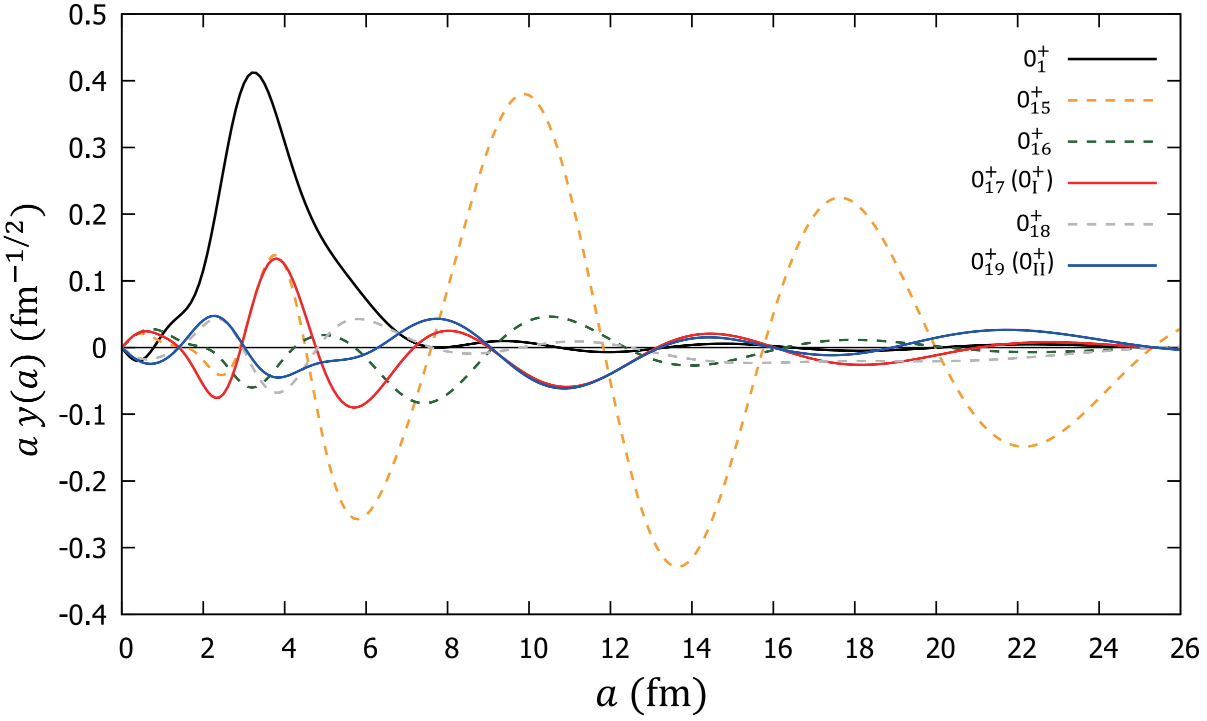

Supplementary Figure 3. The reduced width amplitudes of the ground state and excited states above 5α threshold in ^{20}Ne in the channel of ^{16}O (0_2^+) + α. The ground state 0_1^+, 0_{17}^+ ($0_{\rm I}^+$), and 0_{19}^+ ($0_{\rm II}^+$) states are shown in solid lines. The 0_{15}^+, 0_{16}^+, and 0_{18}^+ states are shown in dashed lines.

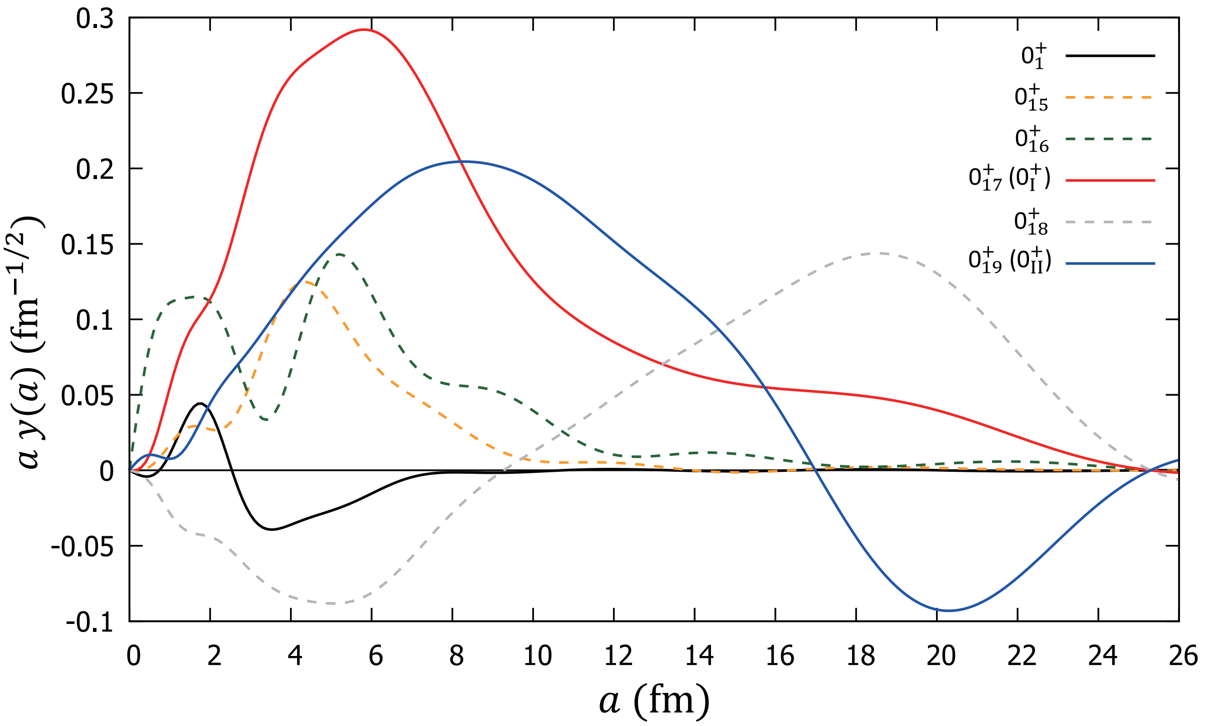

Supplementary Figure 4. The reduced width amplitudes of the ground state and excited states above 5α threshold in ^{20}Ne in the channel of ^{16}O (0^+_6) + α. The ground state 0^+_1, 0^+_{17} (0^+_{I}), and 0^+_{19} (0^+_{II}) states are shown in solid lines. The 0^+_{15}, 0^+_{16}, and 0^+_{18} states are shown in dashed lines.
